# Alternatively Spliced Methionine Synthase in SH-SY5Y Neuroblastoma Cells: Cobalamin and GSH Dependence and Inhibitory Effects of Neurotoxic Metals and Thimerosal

**DOI:** 10.1155/2016/6143753

**Published:** 2016-02-18

**Authors:** Mostafa Waly, Verna-Ann Power-Charnitsky, Nathaniel Hodgson, Alok Sharma, Tapan Audhya, Yiting Zhang, Richard Deth

**Affiliations:** ^1^Department of Food Science and Nutrition, College of Agricultural and Marine Sciences, Sultan Qaboos University, 123 Al-Khoud, Oman; ^2^Natural Sciences Department, Regis College, Weston, MA 02493, USA; ^3^Department of Neurology, Boston Children's Hospital, Boston, MA 02215, USA; ^4^Department of Pharmaceutical Sciences, MCPHS University, Manchester, NH 03101, USA; ^5^Department of Medicine, New York University Medical School, New York, NY 10016, USA; ^6^Department of Pharmaceutical Sciences, Northeastern University, Boston, MA 02115, USA; ^7^Department of Pharmaceutical Sciences, Nova Southeastern University, Fort Lauderdale, FL 33328, USA

## Abstract

The folate and cobalamin (Cbl-) dependent enzyme methionine synthase (MS) is highly sensitive to oxidation and its activity affects all methylation reactions. Recent studies have revealed alternative splicing of MS mRNA in human brain and patient-derived fibroblasts. Here we show that MS mRNA in SH-SY5Y human neuroblastoma cells is alternatively spliced, resulting in three primary protein species, thus providing a useful model to examine cofactor dependence of these variant enzymes. MS activity was dependent upon methylcobalamin (MeCbl) or the combination of hydroxocobalamin (OHCbl) and S-adenosylmethionine (SAM). OHCbl-based activity was eliminated by depletion of the antioxidant glutathione (GSH) but could be rescued by provision of either glutathionylcobalamin (GSCbl) or MeCbl. Pretreatment of cells with lead, arsenic, aluminum, mercury, or the ethylmercury-containing preservative thimerosal lowered GSH levels and inhibited MS activity in association with decreased uptake of cysteine, which is rate-limiting for GSH synthesis. Thimerosal treatment decreased cellular levels of GSCbl and MeCbl. These findings indicate that the alternatively spliced form of MS expressed in SH-SY5Y human neuronal cells is sensitive to inhibition by thimerosal and neurotoxic metals, and lower GSH levels contribute to their inhibitory action.

## 1. Introduction

MS is a multidomain enzyme which transfers a folate-derived methyl group to homocysteine (HCY), thereby creating methionine. The cobalamin (Cbl) cofactor of MS, its Cbl[I] form, directly participates in the transfer reaction by abstracting a folate-derived methyl group, temporarily creating methylcobalamin (MeCbl), and then transferring the methyl group to HCY [1]. However, if Cbl[I] oxidizes prior to MeCbl formation, enzyme activity is temporarily halted, increasing HCY diversion to the transsulfuration pathway and augmenting formation of cysteine, the rate-limiting metabolite for synthesis of the antioxidant GSH [2, 3]. In this manner Cbl serves as a redox sensor whose oxidation leads to increased antioxidant synthesis in proportion to cellular demand. MS inactivation is accompanied by decreased methylation activity, caused by lower levels of the methyl donor SAM and higher levels of the methylation inhibitor S-adenosylhomocysteine (SAH) [4]. Thus MS and Cbl link redox status to methylation status, including methylation of DNA and histones, which regulate gene transcription via epigenetic mechanisms [5].

In a previous study [6] we observed age-dependent alternative splicing of MS mRNA in postmortem human cortex, resulting in deletion of exons contributing to the methylfolate-binding domain, as well as a portion of the cap domain, which normally protects Cbl from oxidation [1, 7]. Deletion of cap domain exons would be expected to increase vulnerability of Cbl to oxidation, potentially altering the relationship between redox and methylation. Alternative splicing of MS mRNA has also been reported in fetal human brain [8] and, more recently, in several cultured cell types [9]. However, the functional significance of alternative splicing remains largely unexplored.

Earlier we showed that MS activity in SH-SY5Y human neuroblastoma cells is stimulated by insulin-dependent growth factor-1 (IGF-1), acting via the PI3 kinase signaling pathway, associated with increased methylation activity [10]. We further showed that metal ions (Cu^+^, Pb^2+^, Al^3+^, and Hg^2+^) and the ethylmercury derivative thimerosal potently blocked IGF-1 stimulation of MS and reduced basal activity to very low or undetectable levels. The latter observation strongly suggested that metals might interfere with the role of Cbl by some undefined mechanism.

In the current study we investigated the functional consequences of alternative splicing on MS activity in SH-SY5Y human neuroblastoma cells. We show that MS mRNA in SH-SY5Y cells is subject to alternative splicing, similar to human brain, and MS inhibition by thimerosal and metal ions is related to their ability to lower GSH levels and, in the case of thimerosal, decrease the level of MeCbl.

## 2. Materials and Methods

### 2.1. Cell Culture

SH-SY5Y and LN-18 cells from American Type Culture Collection were grown in *α*-MEM supplemented with 10% (v/v) fetal bovine serum and 1% (v/v) penicillin-streptomycin-fungizone at 37° in a humidified 5% CO_2_-containing atmosphere.

### 2.2. Drugs and Chemicals

GSCbl was a generous gift from Dr. Nicola Brasch (Kent State University) and was synthesized as previously described [10]. Hydroxocobalamin (OHCbl), MeCbl, and other chemicals were purchased from Sigma-Aldrich.

### 2.3. PCR Analysis of MS mRNA

RNA was extracted from cells using Trizol® reagent. As previously described [6], custom primers were designed for each of the five domains and exons of interest using OligoPerfect*™* Designer (Invitrogen). All primers were designed to have between 50 and 60% GC content, an annealing temperature of around 60°C, and a length of about 20 bases. Primer sets were checked for primer-dimer formations and each primer was specific for its desired template. cDNA synthesis and subsequent PCR amplification were performed using the Cloned AMV First-Strand cDNA Synthesis Kit and Platinum® Taq DNA Polymerase High Fidelity (Invitrogen). All cDNA synthesis runs used 1 *μ*g of RNA as well as random oligo primers. For gel electrophoresis 7 *μ*L of sample was mixed with water and 6x loading dye and run on a precast 10% TBE gel using 1x TBE buffer. The gel was run at 200 V for 90 minutes and then stained with SYBR Safe*™* DNA Gel Stain and incubated for 30 minutes on a shaker. Gels were visualized using a UV transilluminator. Primers to different exons, as well as primers to the housekeeping gene GAPDH, were also used in quantitative real-time PCR experiments, using 600 nM of both forward and reverse primers. 2 *μ*L of cDNA and water was added to SYBR® green PCR master mix from Applied Biosystems. PCR was performed with a Taqman 6600 (Applied Biosystems), using the following thermal parameters: one cycle of 5 min at 95°, 40 cycles of 95° for 15 sec, 60° for 1 minute, and 72° for 45 sec, followed by a final extension of 72° for 5 min. All data was analyzed using the ΔΔCt method.

### 2.4. Methionine Synthase Assay

Cells were suspended in phosphate buffer containing 0.25 M sucrose, disrupted by sonication on ice, and centrifuged at 40,000 g for 30 min at 4°C. Assays were performed with the supernatant under anaerobic conditions, as previously described [11]. The standard reaction mixture contained 100 mM potassium phosphate, pH 7.2, 500 *μ*M homocysteine, 152 *μ*M SAM, 2 mM titanium citrate, 250 *μ*M (6*R,S*)-5-[^14^C]methyltetrahydrofolate (GE Healthcare), 50 *μ*M of the specified cobalamin, and cell extract in a final volume of 1 mL. The reaction was initiated by addition of radiolabeled methylfolate, incubated for 60 min at 37°C, and terminated by heating at 98°C for 2 min. Radiolabeled methionine was separated on a Dowex 1-X8 column, which was eluted with 2 mL of water. Control assays, in which sample enzyme was omitted, served as blanks. Each data point was determined in triplicate, and experiments were replicated three times.

### 2.5. Cellular GSH Levels

Approximately 10^6^ cells were treated as described, washed with ice-cold phosphate buffer, pelleted, and resuspended in 100 *μ*L lysis buffer for 10 min on ice. After centrifugation, the supernatant was transferred to a fresh tube and monochlorobimane (25 mM) and glutathione-S-transferase reagent was added, as provided by a commercial kit (Biovision). After a 30 min incubation at 37°C, fluorescence was read at 380/460 nm. GSH content was determined by comparison with values from a standard curve using freshly prepared GSH. Results were obtained in triplicate and each experiment was repeated three times.

### 2.6. Cysteine Uptake

Cells were grown to confluence in six-well plates and treated for 1 hr with metals or thimerosal (100 nM). Media was aspirated and cells were washed with Hank's balanced salt solution (HBSS). Nonradioactive HBSS was replaced with 600 *μ*L of HBSS containing 10 *μ*M [35S]-cysteine (1 *μ*Ci/mL) and 100 *μ*M DTT, followed by a 5 min incubation. The [35S]-cysteine/HBSS mixture was aspirated and treatment was terminated with 2x washes of ice-cold HBSS. Cells were then lysed, scraped, collected in a microcentrifuge tube, and sonicated for 10 seconds. An aliquot of sonicate was retained for protein estimation and the remainder counted for [35S] content. Cysteine uptake was expressed as nmoles/mg protein.

### 2.7. Cobalamin Analysis

Cbl extraction and HPLC mobile phase selection were based on a previously published method [12]. Extraction was performed under dim-red light due to Cbl light sensitivity. Brain tissues were thawed on ice and a 10% homogenate was prepared. 150 *μ*L of ice-cold absolute ethanol was added to 100 *μ*L of each sample homogenate and incubated for 10 min. Protein precipitates were removed by centrifugation at 10,600 RPM for 3 min at 20°C. The resulting supernatant was evaporated to dryness, resuspended with 300 *μ*L PBS, and passed through a syringe-driven filter (0.22 *μ*m). The Cbl extract was then transferred to a conical microautosampler vial, blown with nitrogen, capped, and kept at 4°C in the autosampler cooling tray, covered by aluminum foil to avoid Cbl degradation. 30 *μ*L of sample was injected into an Agilent Eclipse XDB-C8 (3 × 150 mm; 3.5 *μ*m) and Agilent Eclipse XDB-C8 (4.6 × 12.5 mm; 5 *μ*m) guard column by the autosampler. Samples were eluted using the following step gradient: 0–2 min 0% B, 2–14 min 17% B, 14–19 min 30% B, 24–31 min 58% B, and 31-32 min 100% B, and then equilibrate column with 0% B for 2 min at a flow rate of 0.6 mL/min. Mobile phase A contained 0.1% acetic acid/acetate buffer titrated to pH 3.5 with NH_4_OH. Mobile phase B was acetonitrile containing 0.1% acetic acid. Cbls were measured using electrochemical detection with an ESA CoulArray with BDD analytical cell model 5040 electrochemical detector at an operating potential of 1000 mV. Peak area analysis, based on standard curves generated for each compound, was performed using CoulArray software (version 3.06 ESA analysis program package). Sample Cbl levels were normalized against protein content.

### 2.8. Western Blot

Rabbit-derived anti-MS antibodies were raised against peptide containing amino acids 49–69 of the human HCY-binding domain. Serum obtained from the eighth week bleed was used in a standard western blot protocol with an SH-SY5Y cell lysate using chemiluminescence detection.

### 2.9. Statistical Analysis

Individual measurements were carried out in triplicate, with the exception of PCR studies, which were done in duplicate. Results are expressed as means ± SEM. Differences between treatment groups were evaluated for significance using a *t*-test statistic and *P* < 0.05 was considered significant.

## 3. Results

### 3.1. MS Is Alternatively Spliced in SH-SY5Y Cells

**Figure 1:**
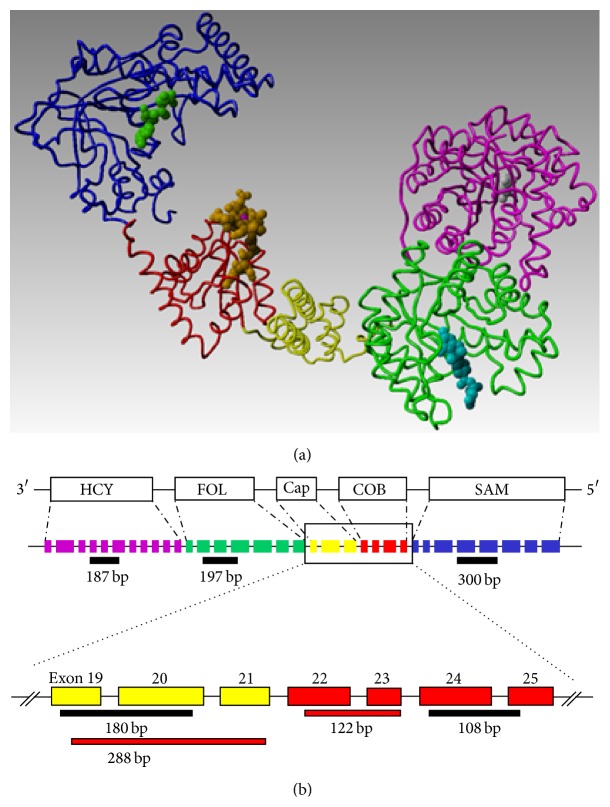
Domain structure and exon composition of cobalamin-dependent MS. MS is comprised of five domains: (a) HCY-binding (pink), methylfolate-binding (green), cap (yellow), cobalamin-binding (red), and SAM-binding (blue). Structures from* E. coli*  and* T. maritima*  (PDB codes 1Q8J, 1K98, and 1MSK, resp.) were used for this composite model. A structurally uncharacterized linker segment between the folate and cap domains is absent. (b) Human MS contains 33 exons specifying the five domains in a sequential manner. PCR sequences used in this study are indicated by red and black line segments.

**Figure 2:**
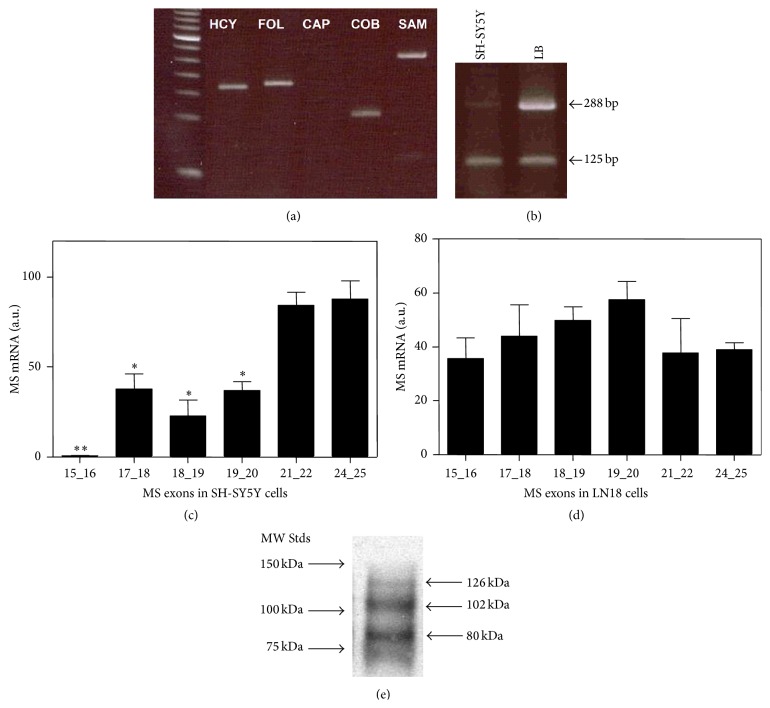
Alternative splicing of MS mRNA in SH-SY5Y cells. (a) Domain-specific PCR with SH-SY5Y cell-derived RNA indicates absence of cap domain products. (b) PCR products with SH-SY5Y and human lymphoblast (LB) RNA. 288 bp product includes exons 19–21; 125 bp product includes only exon 21. (c) qRT-PCR analysis of exon pairs in RNA from SH-SY5Y cells. (d) qRT-PCR analysis of exon pairs in RNA from L18 glioblastoma cells. (e) Western blot analysis of MS in SH-SY5Y cells. ^*∗*^ *P* < 0.05 and ^*∗∗*^ *P* < 0.01 versus untreated group.

We initially examined the status of MS mRNA in SH-SY5Y cells using PCR primer pairs targeted to each of its five domains (Figure 1). As shown in Figure 2(a), cap domain transcripts were not readily visualized, although loading a higher amount of cDNA allowed detection of the predicted full-length (288 bp) cap domain PCR product, along with a smaller 125 bp product, suggestive of alternative splicing (Figure 2(b)). Full-length cap product was robustly detected in RNA from human lymphoblasts, along with a lesser amount of the smaller product. Sequencing of the full-length PCR product confirmed its expected composition (exons 19–21), while the smaller PCR product contained only exon 21. qRT-PCR studies with SH-SY5Y-derived mRNA, using primers directed toward cobalamin-binding (exons 24/25) and cap (exons 19/20) domains, yielded a product ratio of 3.8, significantly different from 1.0 (*P* < 0.02), indicating that approximately 80% transcripts lacked exons 19/20 from the cap domain (data not shown).

**Figure 3:**
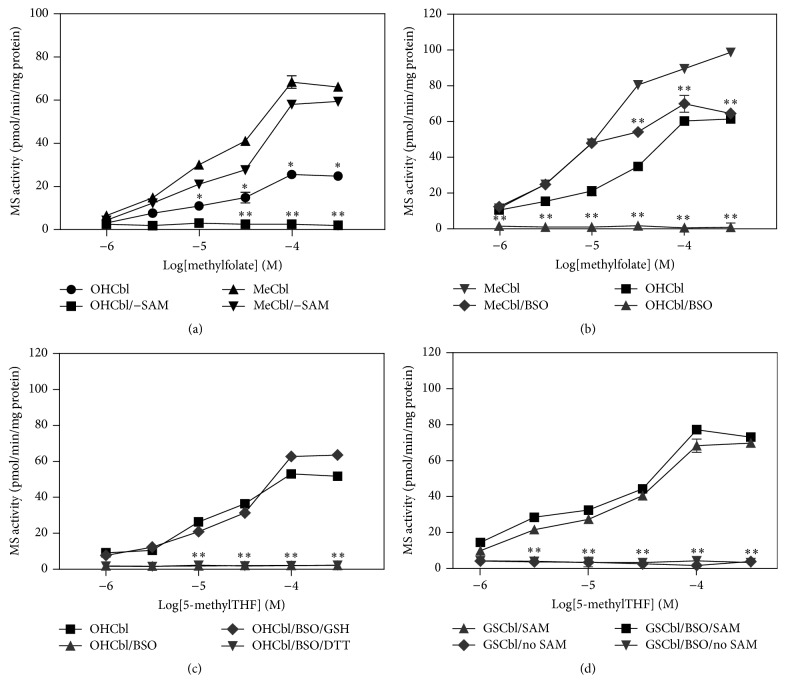
MS activity in SH-SY5Y cells is GSH-dependent and requires MeCbl. (a) Methylfolate-dependent MS activity in the presence of MeCbl or OHCbl ± SAM. ^*∗*^ *P* < 0.01 versus MeCbl. ^*∗∗*^ *P* < 0.01 versus OHCbl/SAM group. (b) Methylfolate-dependent MS activity following inhibition of GSH synthesis with BSO (1 mM; 24 hrs). ^*∗∗*^ *P* < 0.01 versus no BSO treatment. (c) MS activity in the presence of GSH (1 mM) or DTT (1 mM) following BSO treatment. ^*∗∗*^ *P* < 0.01 versus BSO-only. (d) Methylfolate-dependent MS activity in the presence of GSCbl ± SAM ± BSO treatment. ^*∗∗*^ *P* < 0.01 versus untreated group.

To further probe the composition of MS mRNA we designed primers spanning selected splice junctions. Using RNA from SH-SY5Y cells, primers targeted to exons 15/16 did not amplify, while primers for exons 17/18, 18/19, and 19/20 amplified to an intermediate level, as compared to exons 21/22 and 24/25 (*P* < 0.05) (Figure 2(c)). In contrast, using RNA from LN-18 human glioblastoma cells, all primers amplified to a similar extent (Figure 3(d)), indicating that alternative splicing and exon deletion are more extensive in SH-SY5Y neuronal cells. PCR using primers derived from exons 15 and 21 yielded a mixture of two products for SH-SY5Y cells, which upon sequencing were shown to contain exons 15, 19, 20, and 21 or exons 15 and 21. Together these results indicate that exons 16–20 are absent from a majority of MS mRNA transcripts in SH-SY5Y cells, although deletion of exons 16–18 appears to be more extensive than deletion of 19/20. Interestingly, exons 15, 18, and 20 each terminate in CAG, which may facilitate their alternative splicing to exon 21, whereas exons 16, 17, and 19 do not share this canonical sequence.

Consistent with this alternative splicing pattern, western blot analysis using an antibody generated against a HCY-binding domain epitope detected three bands, including a relatively light band at ~126 kDa along with two denser bands of ~102 and ~80 of kDa (Figure 2(e)). Since the calculated MW of the full-length protein is 140.4 kDa, absence of exons 16–18 (16 kDa) may account for the lower density, higher MW band. However, absence of both exons 16–18 (16 kDa) and exons 19 and 20 (8.9 kDa) would yield a MW of ~115 kDa, which is greater than either of the two denser bands, suggesting that one or more additional exons may be absent due to alternative splicing.

### 3.2. MS Activity Is GSH-Dependent

**Figure 4:**
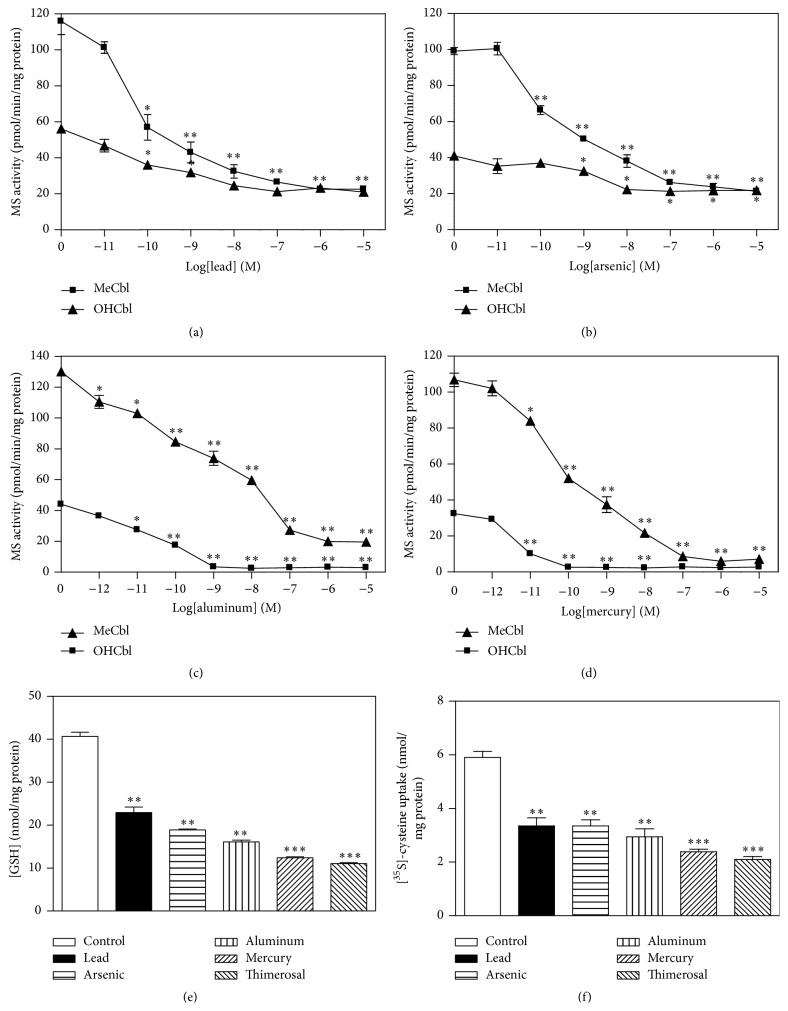
Metal ions and thimerosal inhibit MS activity and decrease levels of GSH in SH-SY5Y cells. Concentration-dependent inhibition of MeCbl and OHCbl-based MS activity by (a) lead, (b) arsenic, (c) aluminum, or (d) mercury. MS activity was measured at 100 *μ*M 5-methylTHF. ^*∗*^ *P* < 0.05 and ^*∗∗*^ *P* < 0.01 versus untreated group. (e) Cellular GSH levels after a 1 hr treatment (100 nM) with lead, arsenic, aluminum, mercury, or thimerosal.   ^*∗∗*^ *P* < 0.01 and ^*∗∗∗*^ *P* < 0.001 versus untreated group. (f) Cellular uptake of cysteine measured after a 1 hr treatment (100 nM) with lead, arsenic, aluminum, mercury, or thimerosal. ^*∗∗*^ *P* < 0.01 and ^*∗∗∗*^ *P* < 0.001 versus untreated group.

MS activity in SH-SY5Y human neuroblastoma cell lysates was assessed by the incorporation of radiolabel from graded concentrations of [^14^C-*methyl*]-methylfolate into methionine under anaerobic conditions, as previously described [11]. MS activity was higher in the presence of MeCbl than OHCbl at all methylfolate concentrations tested (Figure 3(a)). SAM was absolutely required for OHCbl-based, but not MeCbl-based, activity, consistent with SAM-dependent MeCbl formation from OHCbl. Treatment of cells with buthionine sulfoximine (BSO), an inhibitor of GSH synthesis [13], lowered GSH by 67 ± 5% (data not shown) and eliminated OHCbl-based MS activity, measured in the presence of SAM (Figure 3(b)). MeCbl-based activity was partially decreased at higher methylfolate levels. Addition of GSH (1 mM) directly to the MS assay restored OHCbl activity after BSO treatment, although the general reducing agent dithiothreitol (1 mM) failed to restore OHCbl activity (Figure 3(c)), indicating that GSH is specifically required for SH-SY5Y MS activity in this* in vitro* assay. MS activity in the presence of GSCbl was similar to MeCbl-based activity when SAM was provided, but activity was reduced to background in the absence of SAM (Figure 4(d)), indicating that SAM is required for GSCbl to be able to support MS activity. Furthermore, BSO treatment did not interfere with GSCbl-based activity when SAM was provided. Together these observations suggest that MS activity in SH-SY5Y cells requires either MeCbl itself or the capacity to produce MeCbl, either from OHCbl, GSH, and SAM or from GSCbl and SAM.

### 3.3. Inhibition of MS Activity by Metals and Thimerosal

MS activity in SH-SY5Y cells was previously shown to be highly sensitive to metals and the ethylmercury-containing preservative thimerosal [11]. As illustrated in Figures 4(a)–4(d), pretreatment of cells with lead, arsenic, aluminum, or mercury potently inhibited OHCbl- and MeCbl-dependent MS activity, measured after a 60 min incubation. For aluminum and mercury, partial MeCbl-based activity persisted after complete loss of OHCbl-based activity, suggesting a defect in GSH-dependent conversion of OHCbl to MeCbl. Consistent with this possibility, cellular GSH levels were significantly reduced following a 1 hr treatment with each metal at 100 nM (Figure 4(e)). The metal-induced decrease in GSH was associated with a proportionately similar decrease in cellular uptake of cysteine, whose intracellular concentration is rate-limiting for GSH synthesis (Figure 4(f)). Thus metal-induced decreases in GSH can be attributed to a decrease in cysteine uptake, and the decrease in GSH leads to decreased MS activity.

**Figure 5:**
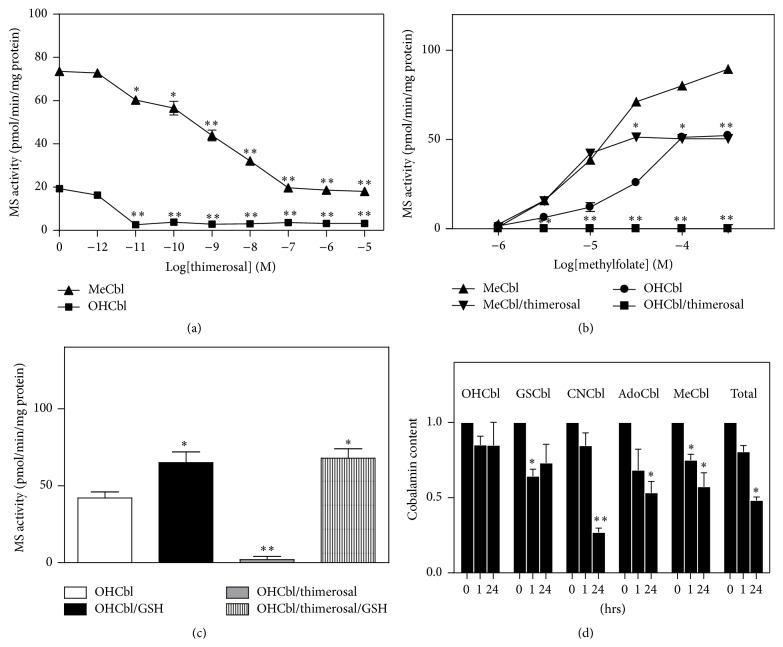
Thimerosal inhibition of MS activity in SH-SY5Y cells. (a) Concentration-dependent inhibition of MeCbl and OHCbl-based MS activity by thimerosal. (b) Thimerosal treatment (10 nM; 1 hr) completely inhibits methylfolate-dependent MS activity in the presence of OHCbl but only partially inhibits activity in the presence of MeCbl. (c) The thimerosal-induced loss of OHCbl-dependent MS activity is reversed by addition of GSH. (d) Cbl levels after thimerosal pretreatment (100 nM) for 4 or 24 hrs. ^*∗*^ *P* < 0.05 and ^*∗∗*^ *P* < 0.01 versus untreated group.

Similar to the effects of metals, thimerosal decreased MS activity in a dose-dependent manner, with OHCbl-based activity being more sensitive than MeCbl-based activity (Figure 5(a)), and this inhibition was associated with decreased GSH (Figure 4(e)) and decreased cysteine uptake (Figure 4(f)). Thimerosal (10 nM) pretreatment completely eliminated MS activity measured in the presence of OHCbl at all concentrations of methylfolate (Figure 5(b)). Activity measured in the presence of MeCbl was unaffected by thimerosal at lower methylfolate concentrations (i.e., ≤10 *μ*M), although the further increase of activity at higher methylfolate levels (i.e., >10 *μ*M) was blocked. This pattern is similar to the effect of BSO on MeCbl-based activity (cf. Figures 3(b) and 5(b)), again suggesting that the inhibitory effect of thimerosal is related to a reduction in GSH. Addition of GSH (1 mM) directly to the MS assay restored OHCbl-based activity after prior thimerosal exposure, confirming that MS inhibition was indeed secondary to lower GSH levels (Figure 5(c)).

The above observations are consistent with the notion that the thimerosal-induced decrease in MS activity reflects lower levels of MeCbl, secondary to decreased GSH levels. To test this possibility, SH-SY5Y cells were treated with 100 nM thimerosal for 1 or 24 hrs and levels of individual Cbl species were measured via an HPLC/electrochemical detection method. As illustrated in Figure 5(d), levels of MeCbl were significantly decreased at both 1 and 24 hrs. GSCbl was significantly decreased at 1 hr and cyanocobalamin (CNCbl), adenosylcobalamin (AdoCbl), and total Cbl were significantly decreased at 24 hrs. These results indicate an important influence of thimerosal on Cbl levels in SY-SY5Y cells which may in part be mediated by a decrease in GSH.

## 4. Discussion

In an earlier study of normal human frontal cortex we found that MS mRNA decreased several hundredfold across the lifespan, accompanied by age-dependent alternative splicing with deletion of exons 19 and 20 [6]. Large-scale sequencing of cDNA from human brain of fetal origin revealed alternatively spliced MS transcripts in which exons 16–18 were deleted [8]. Thus alternative splicing with exon skipping is a normal feature of MS mRNA in human brain, with different patterns occurring at distinct developmental stages. Deletion of exons 16–18 was also recently reported in skin-derived fibroblasts and Caco2 cells [9]. In the current study we demonstrate alternative splicing of MS mRNA in SH-SY5Y human neuronal cells and we further show that MS activity is dependent upon MeCbl, whose formation from OHCbl is associated with an absolute dependence upon the availability of GSH. MS activity is potently inhibited by mercury and other metals which lower GSH levels.

A high proportion of MS mRNA transcripts in SH-SY5Y cells are missing a significant portion of the folate-binding domain (exons 16–18), and a portion of the cap domain (exons 19 and 20) is missing from ~75% of the transcripts. These alternatively spliced transcripts are reflected as lower MW protein species (Figure 3(c)), which can be expected to exhibit distinctive functional properties. Deletion of exons 16–18 would be expected to reduce folate affinity, while deletion of cap domain exons 19 and 20 would be expected to both increase vulnerability of Cbl to oxidation and increase its dissociation. While the pattern of alternative splicing in SH-SY5Y cells overlaps with that of human cerebral cortex, insofar as deletion of exons 16–18 and 19 and 20, lower MW values for denser protein bands in the current study indicate the need for a more extensive and detailed investigation and comparison.

We found alternative splicing to be more prominent in neuronal cells than other cell types, suggesting that GSH-dependent MS activity may be particularly important in the brain, especially with advancing age, when splicing is increased [6]. While the underlying mechanisms regulating this alternative splicing remain unknown, they may be sensitive to changes in redox and oxidative stress status. Thus the neuronal phenotype may provide a more favorable metabolic environment for alternative splicing of MS mRNA, but additional factors, acting through redox and/or methylation pathways, may dictate the extent of splicing, which could vary in a continuous manner across the lifespan.

**Figure 6:**
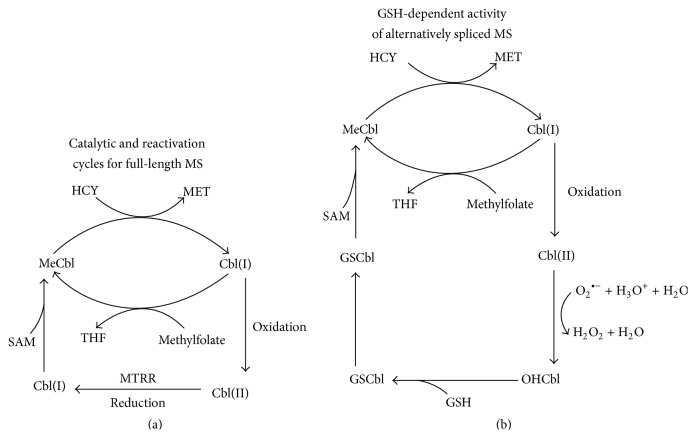
Proposed mechanism for GSH-dependent reactivation of MS activity. During primary turnover, MS carries out methylation of HCY using methylfolate-derived methyl groups which are transiently bound to cobalamin as MeCbl (a). Depending upon redox conditions, the Cbl(I) state can oxidize to Cbl(II), inactivating the enzyme. Reactivation requires either reduction of Cbl(II) to Cbl(I) by methionine synthase reductase (MTRR), followed by SAM-dependent methylation of Cbl(I) to form MeCbl (a), or GSH-dependent formation of MeCbl via conversion of Cbl(II) to OHCbl (b). The latter GSH-dependent mechanism may be associated with alternatively spliced forms of MS for whom the probability of oxidation is enhanced.

The ability of GSCbl, but not OHCbl, to support MS activity in the presence of SAM after GSH depletion strongly suggests that GSH is required for conversion of OHCbl to GSCbl, which is subsequently converted to MeCbl in the presence of SAM. OHCbl is readily converted to GSCbl in the presence of GSH [11, 14, 15], although the physiological role of GSCbl remains to be fully explored. As illustrated in Figure 6(b), we propose that, for a portion of MS that is alternatively spliced, oxidized Cbl(II) is converted to OHCbl by reaction with superoxide anion in a dismutase reaction [16], followed by SAM-dependent methylation to MeCbl. This reactivation sequence implies that activity of alternatively spliced forms of MS is more dependent upon ambient levels of GSH.

Methylmalonic aciduria and homocystinuria type C protein (MMACHC) may be involved in GSH-dependent MS reactivation. Fofou-Caillierez et al. [9] recently demonstrated that both full-length and alternatively spliced forms of MS are complexed with MMACHC, and human MMACHC carries out GSH-dependent dealkylation of MeCbl to yield OHCb [17]. This corresponds to the reverse reaction in Figure 6, raising the possibility that MMACHC may reversibly catalyze interconversion of MeCbl and OHCbl via GSCbl. However, further studies are required to elucidate the potential involvement of MMACHC in MS reactivation.

In an earlier study we showed that lead, aluminum, mercury, and thimerosal are highly potent in their ability to inhibit MS and folate-dependent phospholipid methylation in SH-SY5Y cells [11]. The current results reveal that their inhibition, as well as that of arsenic, is due to a reduction in GSH, secondary to decreased cysteine uptake. Cysteine uptake in SH-SY5Y is primarily mediated by excitatory amino acid transporter 3 (EAAT3) [18], and the ability of mercury to inhibit EAAT3 in primary neurons has been previously reported [19]. Both lead and mercury cause* in vivo* upregulation of EAAT3 expression [20, 21]. Thimerosal decreased MeCbl levels after 4 and 24 hrs, which could also contribute to decreased MS activity.

Autism is a neurodevelopmental disorder associated with low levels of GSH in blood [22–25] and brain [26–28]. We previously reported that frontal cortex MS mRNA levels are decreased in autism [6], and more recently we found that total Cbl and MeCbl levels are 2- to 3-fold lower in frontal cortex of autistic subjects, in association with decreased MS activity [29]. Together with studies showing abnormal DNA methylation status [30–33], these findings support a “Redox/Methylation Hypothesis of Autism” [12], which proposed that the combined impact of environment factors on GSH leads to decreased MS activity with epigenetic consequences that interrupt the normal trajectory of brain development. Inhibitory effects of lead, aluminum, mercury, and arsenic, as well as the pharmaceutical preservative thimerosal on MS activity and Cbl status, are consistent with their recognized activity neurodevelopmental toxins, and impaired methylation, secondary to GSH depletion, is likely to be an important mechanism of their neurotoxicity. Notably, MeCbl treatment has been shown to least partially correct redox and methylation abnormalities in autism [17, 34], which, in some cases, is associated with cognitive improvement [35].

Our findings are subject to several important limitations. While SH-SY5Y cells are the mostly widely utilized cell culture model for human neurons, they are a transformed cell line derived from a peripheral neuroblastoma tumor [33]. As such, their properties are clearly different from primary human neurons, including a difference in MS splicing. Moreover, cell culture conditions do not mirror those found* in vivo*, which could significantly affect our results. Finally, the concentrations of metal ions which occur in human brain have not been directly measured, so the extent to which MS is affected by them under* in vivo* conditions remains unknown. Caution must be exerted in extrapolating our findings to the brain.

In summary, we demonstrate that alternative splicing of MS mRNA is associated with GSH dependence in cultured human neuronal cells, and substances which lower GSH levels, such as lead, arsenic, aluminum, mercury, and thimerosal, inhibit MS activity with high potency. Additional studies are needed to further investigate the possible significance of glutathione-dependent MS activity in the brain and to evaluate the potential benefit of MeCbl or GSCbl in treating neurological, neuropsychiatric, and neurodegenerative disorders.
